# Supplementary material for: Human FBXL8 Is a Novel E3 Ligase Which Promotes BRCA Metastasis by Stimulating Pro-Tumorigenic Cytokines and Inhibiting Tumor Suppressors
Source: Cancers (Basel). 2020 Aug 7;12(8):2210. doi: 10.3390/cancers12082210 (PMC7465060; doi:10.3390/cancers12082210)
Supplement: Supplementary file 1 [file cancers-12-02210-s001.pdf]

Supplementary Materials:

Human FBXL8 Is a Novel E3 Ligase Which Promotes BRCA Metastasis by Stimulating Pro-Tumorigenic Cytokines and Inhibiting Tumor Suppressors

Samples / Gene	Patient 1		Patient 2		Patient 3		Patient 4		Patient 5	
	Normal	Carcinorma	Normal	Carcinorma	Normal	Carcinorma	Normal	Carcinorma	Normal	Carcinorma
SKP1	579	594	598	873	473	571	365	269	540	643
SKP2	16	35	11	30	11	15	19	21	15	31
CUL1	45	72	80	112	75	68	78	96	42	79
FBXW7	60	80	61	113	31	41	41	27	55	70
SAG	48	81	54	77	43	32	44	68	44	73
FBXO4	29	31	35	23	10	22	13	23	26	41
RBX1	56	78	49	52	49	37	54	63	50	58

Figure S1. The up-regulation of key members of SCF components in BRCA tissues. Log_2. fold change of SCF components identified from the global RNA sequencing. Key factors highlighted include: SKP1 (S-phase kinase-associated protein 1), SKP2, CUL1 (cullin 1), FBXW7 (F-box/WD repeat-containing protein 7), SAG (Sensitive to apoptosis gene), FBXO4 (F-box only protein 4) and RBX1 (ring-box 1). The significant actions of SCF components overexpression in breast carcinoma tissues are shown in red. Numbers in red indicate increase in expression of the gene (log_2 fold change) in BRCA tissues.

Patient	6	7	8	9	10	11	12	13	14	15	16	17	18	19	20
Diagnosis	IDC	IDC	ILC	IDC	IDC	IDC	IDC	ILC	IDC	IDC	IDC	IDC	IDC	MC	IDC
Histologic type	DCIS	IMC	DCIS	IMC	IMC	IMC	IMC	IMC	IDC	IDC	IDC	IMC	IDC	MC	IDC
Histologic grade	3	3	3	1	1	2	2	2	1	2	3	2	2	1	1
pT	Tis	T1	T1b	T1	T1c	T1a	T1	T1c	T1b	T1c	T1c	T2	T1c	T1b	T1c
pN	N0	N0	N0	N0	N0	N0	N0	N0	N0	N0	N0	N0	N0	N0	N0
pM	M0	M0	M0	M0	M0	M0	M0	M0	M0	M0	M0	M0	M0	M0	M0
Stage	0	IA	IA	IA	IA	IA	IA	IA	IA	IA	IA	IA	IA	IA	IA
ER	-	+	-	+	+	-	+	+	+	+	-	-	+	+	+
PR	+	+	+	+	+	-	-	+	+	+	-	-	+	+	-
HER2	3+	2+	3+	2+	2+	3+	3+	2+	1+	2+	2+	1+	1+	1+	2+
Ki67	NA	10%	NA	15%	5%	10%	25%	15%	10%	60%	30%	50%	60%	3%	10%
Age (year)	50	57	48	62	53	68	45	70	41	43	55	50	51	56	50
Gender (F/M)	F	F	F	F	F	F	F	F	F	F	F	F	F	F	F
FBXL8 Tumor (fc)	1.9?	0.1?	2.1?	2.2?	1.3?	0.5?	1.5?	1.9?	0.5?	1.8?	<0.01?	0.4?	1.6?	1.5?	1.5?

Patient	21	22	23	24	25	26	27	28	29
Diagnosis	ILC	IDC	IDC	IDC	IDC	IDC	IDC	IDC	IDC
Histologic type	ILC	IMC	IMC	IMC	IDC	IDC	IDC	IDC	MMC
Histologic grade	2	2	1	3	2	2	2	NA	1
pT	T2	T2	T2	T1c	T2	T1c	T1c	T2	T2
pN	N0	N0	N0	N0	N0	N1	N1	N1	N1a
pM	M0	M0	M0	M0	M0	M0	M0	M0	M0
Stage	IIA	IIA	IIA	IIA	IIA	IIA	IIA	IIB	IIB
ER	+	+	-	+	+	-	+	-	+
PR	+	+	-	+	+	-	+	-	+
HER2	1+	1+	0	1+	1+	1+	2+	3+	2+
Ki67	15%	30%	3%	75%	30%	50%	20%	20%	10%
Age (year)	62	49	65	66	54	54	38	72	45
Gender (F/M)	F	F	F	F	F	F	F	F	F
FBXL8 Tumor (fc)	1.9?	2.4?	2.4?	<0.01?	3.3?	0.1?	1.1?	1.2?	2.5?

Patient	30	31	32	33	34	35	36	37
Diagnosis	IDC	IDC	IDC	IDC	IDC	IDC	IDC	IDC
Histologic type	IMC	IDC	IDC	IDC	IMC	IMC	IMC	IMC
Histologic grade	3	3	3	2	2	2	2	2
pT	T1c	T3	T2	T1c	T4b	T4b	T2	T2
pN	N2a	N1	N2a	N2a	N2a	N1a	N3a	N3a
pM	M0	M1	M0	M0	M0	M0	M0	M0
Stage	IIIA	IIIA	IIIA	IIIA	IIIB	IIIB	IIIC	IIIC
ER	+	+	-	+	+	+	+	+
PR	+	+	-	+	+	+	+	+
HER2	3+	2+	3+	3+	1+	2+	1+	0
Ki67	35%	80%	50%	40%	30%	30%	20%	10%
Age (year)	44	51	42	50	49	63	56	42
Gender (F/M)	F	F	F	F	F	F	F	F
FBXL8 Tumor (fc)	1.3?	1.9?	3.4?	1.1?	4.5?	3.1?	1.3?	0.7?

Figure S2. Clinicopathological parameters in paired primary breast carcinoma tissues. To affirm the RNA-seq data, we acquired more primary tissues for the qRT-PCR analysis. Clinicopathological information provided from TMUH, includes diagnosis, histological type, histological grade, pTNM Pathological Classification, stage, ER, PR, HER2, Ki67, age and gender in respective tissue. Paired primary tissues from breast carcinoma (*n* = 32) and corresponding normal breast tissues (*n* = 32) were examined. Tissue samples are categorized into three groups based on cancer staging. (**A**): 0/IA as initial stage (*n* = 30, including *n* = 15 carcinoma tissues and *n* = 15 corresponding normal breast tissues); (**B**): IIA/IIB as middle stage (*n* = 18, including *n* = 9 carcinoma tissues and *n* = 9 corresponding normal breast tissues) and (**C**): IIIA/IIIB/IIIC as late stage (*n* = 16, including *n* = 8 carcinoma tissues and *n* = 8 corresponding normal breast tissues). The mRNA levels (fold change) of FBXL8 are highlighted - red arrow (↑) and blue arrow (↓) indicate upregulation and downregulation, respectively, of the FBXL8 gene expression. F, female; IMC, Invasive mammary carcinoma; IDC, Invasive ductal carcinoma; ILC, Invasive lobular carcinoma; MC, Mucinous carcinoma; MMC, Mixed mucinous carcinoma; DCIS, Ductal carcinoma in situ; fc, fold change; ER, estrogen receptor; PR, progesterone receptor; pN, pathologic lymph node status; pT, Primary Tumour; pM, Distant Metastasis.

Patient	38	39	40	41	42	43	44	45	46
Diagnosis	IDC	IDC	IDC	IDC	IDC	IDC	IDC	IDC	IDC
Histologic type	IDC	IDC	IDC	IDC	IDC	IDC	IDC	IDC	IDC
Histologic grade	1	2	3	3	1	2	3	3	3
pT	T1c	T1b	T1c	T1c	T1c	T1c	T1c	T1c	T1c
pN	N0	N0	N0	N0	N0	N0	N0	N0	N1
pM	M0	M0	M0	M0	M0	M0	M0	M0	M0
Stage	IA	IA	IA	IA	IA	IA	IA	IA	IB
ER	+	-	+	+	+	+	+	-	-
PR	+	-	-	+	+	+	-	-	-
HER2	2+	0	2+	2+	2+	2+	3+	0	2+
Ki67	10%	25%	15%	10%	15%	50%	20%	70%	10%
Age (year)	59	30	46	63	60	71	51	51	58
Gender (F/M)	F	F	F	F	F	F	F	F	F

Patient	47	48	49	50	51	52	53	54	55	56	57
Diagnosis	IDC	IDC	IDC	IDC	IDC	IDC	IDC	IDC	IDC	IDC	IDC
Histologic type	IDC	IDC	IDC	IDC	IDC	IDC	IDC	IDC	IDC	IDC	IDC
Histologic grade	2	3	3	3	2	1	2	3	2	2	3
pT	T2	T2	T1c	T2	T2	T1c	T2	T2	T2	T2	T2
pN	N0	N0	N1	N0	N0	N1	N1	N1	N1	N1	N1
pM	M0	M0	M0	M0	M0	M0	M0	M0	M0	M0	M0
Stage	IIA	IIA	IIA	IIA	IIA	IIA	IIB	IIB	IIB	IIB	IIB
ER	-	+	-	-	-	+	+	+	+	+	-
PR	-	+	-	-	-	-	+	+	+	+	-
HER2	1+	1+	3+	1+	0	1+	2+	0	1+	0	3+
Ki67	25%	40%	40%	50%	75%	5%	10%	40%	5%	5%	50%
Age (year)	60	39	46	56	51	48	66	80	51	51	56
Gender (F/M)	F	F	F	F	F	F	F	F	F	F	F

Patient	58	59	60	61	62	63	64	65	66	67
Diagnosis	IDC	IDC	IDC	IDC	IDC	IDC	IDC	IDC	IDC	IDC
Histologic type	IDC	IDC	IDC	IDC	IDC	IDC	IDC	IDC	IDC	IDC
Histologic grade	2	2	3	3	3	2	2	2	3	3
pT	T3	T2	T2	T3	T2	T2	T2	T2	T3	T3
pN	N2	N2	N1	N2	N2	N2	N2	N2	N2	N2
pM	M0	M0	M0	M0	M0	M0	M0	M0	M0	M0
Stage	IIIA	IIIA	IIIA	IIIA	IIIA	IIIA	IIIA	IIIA	IIIA	IIIA
ER	+	+	-	-	+	+	-	+	+	+
PR	+	+	-	-	+	+	-	+	+	+
HER2	1+	3+	1+	0	2+	1+	0	0	2+	0
Ki67	50%	65%	40%	60%	8%	20%	70%	30%	50%	30%
Age (year)	42	57	74	50	72	38	72	51	34	70
Gender (F/M)	F	F	F	F	F	F	F	F	F	F

Figure S3. Clinicopathological parameters in primary BRCA tissues used for IHC. analysis**.** To affirm the transcriptional data, we retrospectively studied primary breast tissues based on the IHC analysis. Clinicopathological information provided from TMUH, include diagnosis, histological type, histological grade, pTNM Pathological Classification, stage, ER, PR, HER2, Ki67, age and gender in respective tissues. A total 60 primary tissues were examined, constituting breast carcinoma (n = 30) and paired normal breast tissues (n = 30). Samples were categorized into three groups based on cancer staging. (**A**): IA/IB as initial stage (n = 18, including n = 9 carcinoma tissues and n = 9 normal breast tissues); (**B**): IIA/IIB as middle stage (n = 22, including n = 11 carcinoma tissues and n = 11 normal breast tissues) and (**C**): IIIA as late stage (n = 20, including n = 10 carcinoma tissues

and $n = 10$ normal breast tissues). The mRNA levels (fold change) of FBXL8 are highlighted here. Red arrow (↑) and blue arrow (↓) indicate upregulation and downregulation of FBXL8 gene expression, respectively. F, female; IMC, Invasive mammary carcinoma; IDC, Invasive ductal carcinoma; ILC, Invasive lobular carcinoma; MC, Mucinous carcinoma; MMC, Mixed mucinous carcinoma; DCIS, Ductal carcinoma in situ; fc, fold change; ER, estrogen receptor; PR, progesterone receptor; pN, pathologic lymph node status; pT, Primary Tumour; pM, Distant Metastasis.

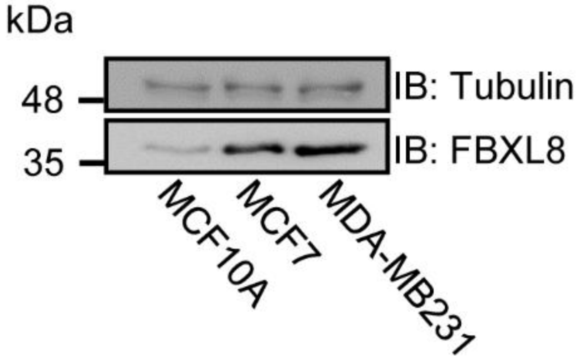

Figure S4. Endogenous levels of FBXL8 in BRCA cells and control MCF10A cells. The endogenous levels of FBXL8 protein were examined in MCF7 and MDA-MB231 cells. MCF10A, a non-carcinoma cell line, was also be examined. FBXL8 was highly-expressed in both MCF7 and MDA-MB231 cells. On the other hand, MCF10A cells only expressed low/basal level of FBXL8 compared to MCF7 and MDA-MB231 cells.

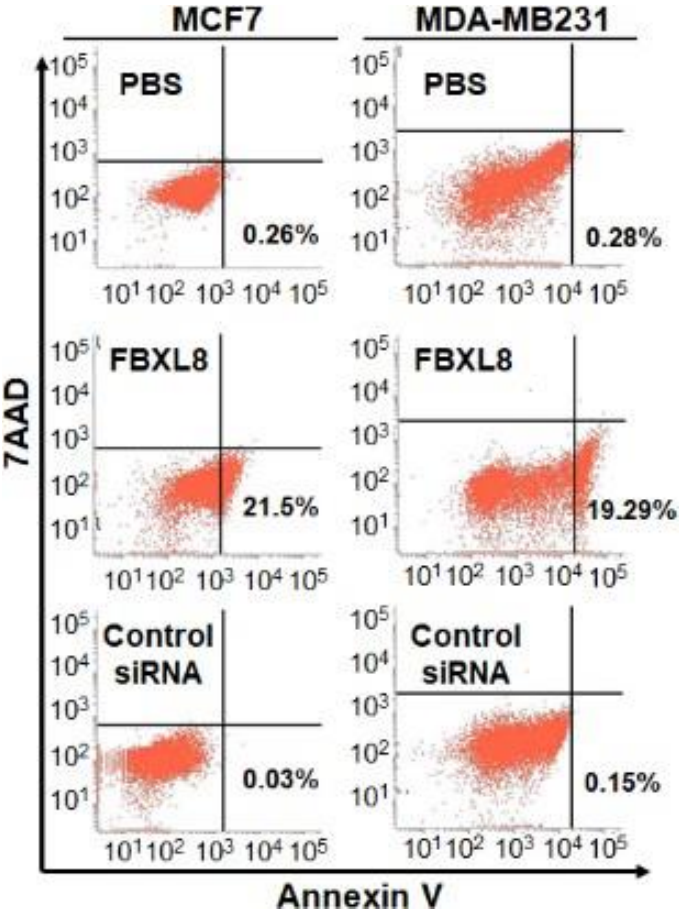

Figure S5. FACS analysis of apoptosis after siRNA transfection treatments of breast cancer cells. Representative histograms of cell apoptosis assay (Annexin V and 7AAD double staining), including

controls (PBS and control-siRNA). Two breast cancer cell lines are tested, including MCF7 and MDA-MB231. Early apoptotic cells are indicated by Annexin V^{+}/$7AAD^{-}$ (shown as %).

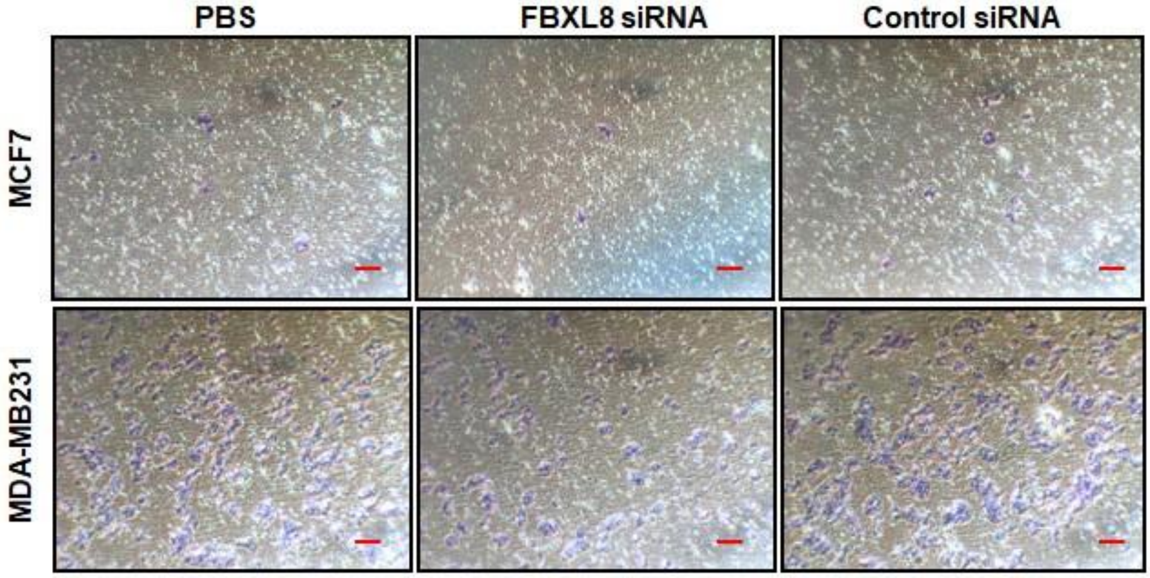

Figure S6. Representative microscopy images of breast cancer cell invasion. 24 h after treatment of breast cancer cells with FBXL8 siRNA, control siRNA or PBS control, matrigel invasion assay was performed. FBXL8 siRNA efficiently suppressed breast cancer invasion. Two breast cancer cell lines are tested, including MCF7, and MDA-MB231. After another 24h incubation, cells that had migrated from the upper to the lower side of the filter were imaged and counted with a light microscope (5 fields/filter). Scale bar is 100 μm, shown as the red color line (—).

A. STRING

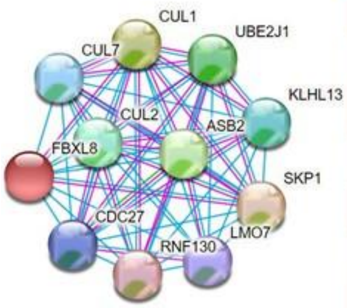

For FBXL8	Score
SKP1	0.937
CUL1	0.901
ASB2	0.900
UBE2J1	0.900
CUL2	0.900
KLHL13	0.900
CUL7	0.900
CDC27	0.900
LMO7	0.900
RNF130	0.900

C

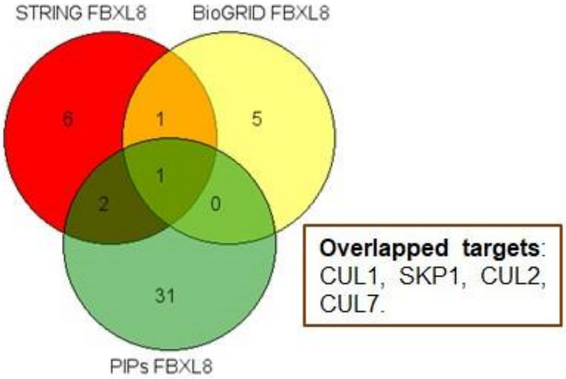

B. BioGRID and PIPs

Database	Predicted targets
**BioGRID**	SCF E3-associated: SKP1, CUL1, UBE2M.<br>DNA binding protein: ORC4, SNAI1.<br>Others: HSP90AA1, CHMP6.
**PIPs**	SCF E3-associated: CUL2, PARC, CUL5, CUL1, CUL7, RNF8, RNF31, ANAPC2 .<br>DNA binding protein: SMAD1, SMAD2, SMAD3, SMAD4, IRF3, IRF5, IRF8, NBN.<br>Cyclin family: CCNA2, CCNB1, CCNB2, CCND2, CCND3, CCNG1, CCNL1, CCNL2, CCNC, CCNF, CCNI, CCNH, CCNE1, CCNE2, C10orf9, CDKN2B.<br>Others: CNT2.

Figure S7. Computer modeling prediction of FBXL8-associated proteins. Several database are used to search the potential proteins associated with FBXL8, including STRING, BioGRID and PIPs (**A**) shows predicted factors by STRING may involve in FBXL8-binding. (**B**) shows summarized list of FBXL8-binding proteins based on BioGRID and PIPs. (**C**) shows protein targets either present in any two database or in allthree databases, including CUL1, SKP1, CUL2 and CUL7.

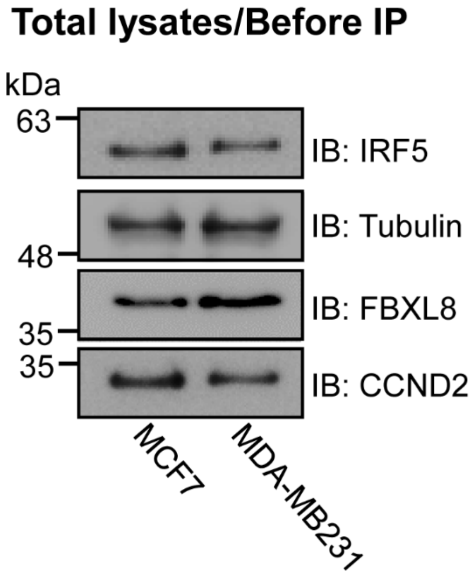

Figure S8. Immunoblotting analysis of whole lysates of BRCA cell. Western blot analysis of whole cell lysates shows the presence of IRF5, FBXL8, and CCND2 in the MCF7 and MDA-MB231 cells (before IP). Tubulin was used as a loading control.

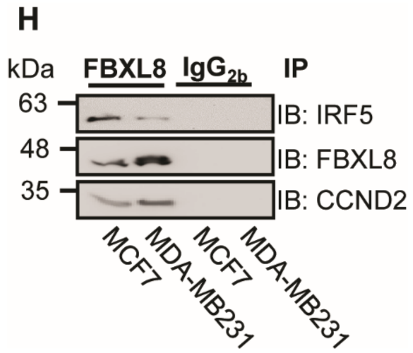

Figure S9. Original images of Figure 6H for blots.

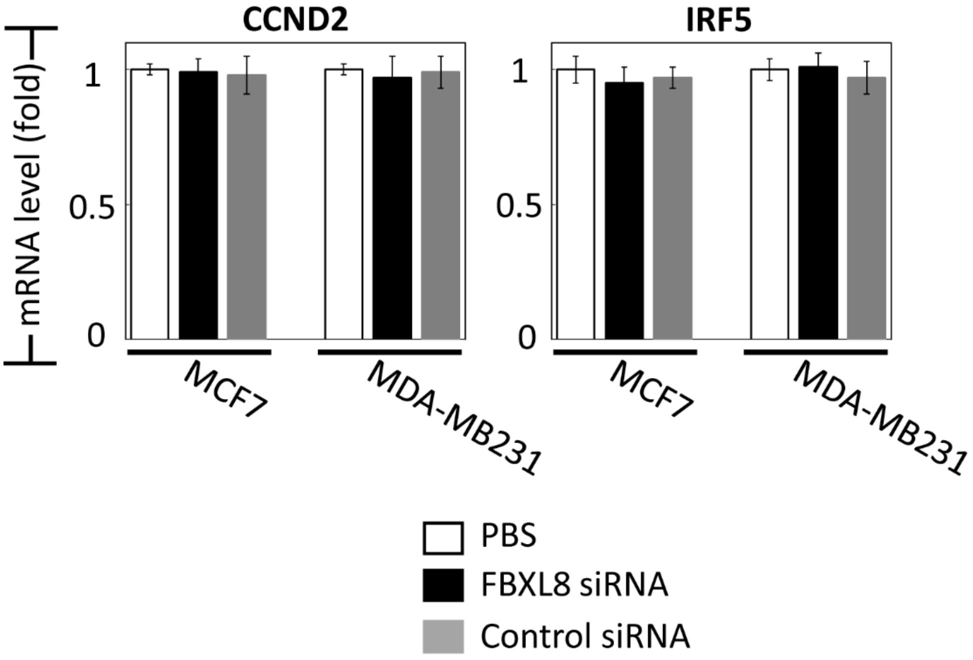

Figure S10. Transcript levels of CCND2 and IRF5 in BRCA cells upon knockdown of FBXL8 by siRNA treatment. 48 h after treatment of the MCF7 and MDA-MB231 cells with FBXL8-siRNA or control siRNAs, qRT-PCR was carried out to determine the mRNA levels of cancer suppressors, CCND2 and IRF5. The transcript levels of CCND2 and IRF5 remained unchanged when FBXL8 was knocked down. Data are presented as means ±SD ($n = 3$).

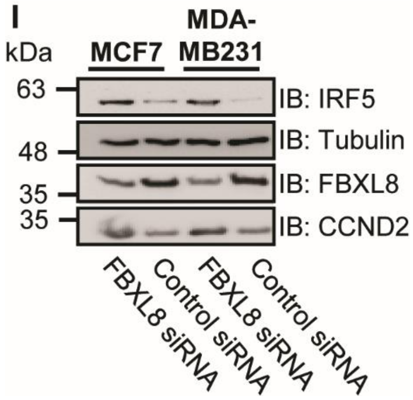

Figure S11. Original images of Figure 6I for blots.
